# Mechanical Behaviour of Photopolymer Cell-Size Graded Triply Periodic Minimal Surface Structures at Different Deformation Rates

**DOI:** 10.3390/ma17102318

**Published:** 2024-05-14

**Authors:** Yunus Emre Yılmaz, Nejc Novak, Oraib Al-Ketan, Hacer Irem Erten, Ulas Yaman, Anja Mauko, Matej Borovinsek, Miran Ulbin, Matej Vesenjak, Zoran Ren

**Affiliations:** 1 Faculty of Mechanical Engineering, University of Maribor, 2000 Maribor, Slovenia; anja.mauko@um.si (A.M.); matej.borovinsek@um.si (M.B.); miran.ulbin@um.si (M.U.); matej.vesenjak@um.si (M.V.); zoran.ren@um.si (Z.R.); 2 Core Technology Platforms, New York University Abu Dhabi, Abu Dhabi P.O. Box 129188, United Arab Emirates; oga2@nyu.edu; 3 Department of Mechanical Engineering, Faculty of Engineering, İzmir Institute of Technology, Gülbahçe, Urla, İzmir 35347, Türkiye; erteniremm@gmail.com; 4 Department of Mechanical Engineering, Faculty of Engineering, Middle East Technical University, Ankara 06800, Türkiye; uyaman@metu.edu.tr

**Keywords:** cellular materials, triply periodical minimal surface, photopolymer, mechanical properties, strain rate, experimental compressive testing, computer simulations

## Abstract

This study investigates how varying cell size affects the mechanical behaviour of photopolymer Triply Periodic Minimal Surfaces (TPMS) under different deformation rates. Diamond, Gyroid, and Primitive TPMS structures with spatially graded cell sizes were tested. Quasi-static experiments measured boundary forces, representing material behaviour, inertia, and deformation mechanisms. Separate studies explored the base material’s behaviour and its response to strain rate, revealing a strength increase with rising strain rate. Ten compression tests identified a critical strain rate of 0.7 s^−1^ for “Grey Pro” material, indicating a shift in failure susceptibility. X-ray tomography, camera recording, and image correlation techniques observed cell connectivity and non-uniform deformation in TPMS structures. Regions exceeding the critical rate fractured earlier. In Primitive structures, stiffness differences caused collapse after densification of smaller cells at lower rates. The study found increasing collapse initiation stress, plateau stress, densification strain, and specific energy absorption with higher deformation rates below the critical rate for all TPMS structures. However, cell-size graded Primitive structures showed a significant reduction in plateau and specific energy absorption at a 500 mm/min rate.

## 1. Introduction

The unique mechanical properties of cellular materials can be attributed to their distinctive pore structures characterised by morphology and topology. Morphology refers to the shape and arrangement of the pores, while topology refers to how the pores are connected and organised. These distinctive pore structures contribute to the unique mechanical properties of cellular materials. The morphology of cellular material can be either open or closed [1], and the topology can be either periodic or stochastic [2]. The pores are arranged in a regular, recurring pattern in periodic cellular materials. They can be further classified into three main subtypes: strut-based, sheet-based, and plate-based lattices, based on the geometry of their pores and how they are connected [3,4].

Triply Periodic Minimal Surfaces (TPMSs) are a specific type of sheet-based cellular structures that have gained increasing attention due to their superior mechanical properties and adjustable geometry. The mathematical foundation of TPMS design allows for a flexible approach to their optimisation and design, making them ideal for various engineering applications [4], particularly in high strain rate loading conditions [5,6,7,8]. TPMSs comprise continuous, smooth, non-self-intersecting surfaces, which minimise stress concentration during deformation [9].

Studies have shown that these structures have a superior strength-to-weight ratio [7] because of their unique deformation behaviour. Functionally graded cellular structures are at the forefront of material development [8,9,10], offering customisable graded parameters such as density, wall thickness, or parametric geometry. Recent investigations into functionally graded cellular materials, as documented in references [11,12], have unveiled significant insights, demonstrating the potential to tailor graded parameters and optimize various functional properties. Notably, these studies highlight enhancements in shock-absorbing capacity and reaction force under specific loads. Furthermore, Zhang [12] notes that the highest energy absorption capacity is achieved by keeping density at its maximum on the side close to the load and linearly decreasing it towards the support area away from the load.

The mechanical properties of cellular materials are strongly influenced by the raw materials used to fabricate them [13]. For example, using photopolymer to build TPMS structures affects stiffness, strength [14], and energy absorption capacity [15]. To fully understand and optimise the mechanical behaviour of TPMS structures, it is essential to investigate the raw material’s direct effects on the structure’s overall performance. This can be achieved through experimental testing and computational modelling, which can provide insights into the material properties and deformation behaviour of the TPMS structure.

Additive manufacturing (AM) allows for precise control over engineered materials’ morphological and topological properties, making it possible to fabricate complex and customised structures, such as cellular structures [3]. Stereolithography (SLA) is an AM method that enables the production of very fine and high-resolution parts, including TPMS structures. With the wide range of photopolymer options available, complex cellular structures can be produced with decent resolution, making SLA a popular choice for TPMS fabrication [16]. The ability of SLA to achieve excellent surface finishes and intricate geometries makes it an ideal method for fabricating TPMS structures that are functional, aesthetically pleasing, and customised to the application’s specific requirements.

Much research is focused on the experimental characterisation of novel cellular structures to understand their mechanical behaviour [15]. The characterisation of fundamental material properties is typically accomplished through quasi-static experiments using compression testing apparatus [16,17,18]. Conducting mechanical characterisations at higher strain rates [19,20,21] is crucial for the comprehensive characterisation of advanced cellular materials. In addition to experimental material characterisation, topology analysis of cellular metamaterials is vital for understanding their unique structural arrangements and optimising their mechanical properties [17,22,23,24]. Advanced additive manufacturing of cellular materials enables the fabrication of complex geometries and customised internal architectures, leading to enhanced mechanical performance and functional capabilities [20,21,22]. Using high-accuracy sensors with high-frequency data acquisition systems has enabled precise capture of boundary forces [25,26]. However, measuring boundary forces involves the effects of various factors such as base material strain rate sensitivity, inertia, cell grading, morphology, and topology, making it challenging to extract the specific influence of each parameter.

This study aims to separate the effects of base material and inertia by conducting computational and experimental investigations. Specifically, the mechanical behaviour of three different TPMS morphologies (Diamond, Gyroid, and Primitive) with cell grading along the axial direction and made of the photopolymer base “Grey Pro” were studied at various deformation rates. The shells’ actual connectivity was observed using an X-ray microscopy machine to gain insight into the structures’ topology properties. A full-field deformation analysis using digital image correlation was conducted to examine non-homogeneous deformation. By comprehensively characterising the mechanical properties of TPMS structures, this study seeks to provide valuable insights for designing and optimising such structures for various applications.

## 2. Materials and Methods

### 2.1. Design of Proposed Samples

The in-house-developed software MSLattice (https://github.com/MSLattice/MSLattice_windows, accessed on 7 May 2023) [27] was used to design the samples. The software is based on level-set equations commonly used to describe minimal surfaces and control their topology. Three unit cells of TPMS structures were considered in this study: Diamond, Gyroid, and Primitive. Detailed descriptions of these unit cells can be found in Ref. [28].

**Figure 1:**
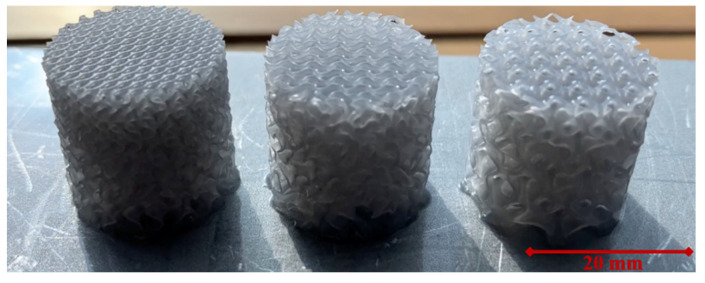
Fabricated cell-size graded Diamond, Gyroid, and Primitive samples.

Cylindrical samples were employed to evaluate production feasibility and facilitate comprehensive mechanical characterisation. The design objective was to minimise sample dimensions to reduce production time and cost. Additionally, the sample size should not exceed the dimensions of the experimental apparatus used to load the specimens. However, sufficient unit cells were required to achieve substantial strains and superior energy absorption characteristics. Considering these criteria, samples of various sizes and dimensions were evaluated, and those deemed most favourable for manufacturability and superior performance in subsequent mechanical characterisations were selected. The selected samples had a relative density of 16%, a radius of 10 mm, a height of 20 mm, a 3 mm unit cell size on one side, and a 6 mm unit cell size on the other side. Designed parts were fabricated using Stereolithography (SLA) with Grey Pro Resin (Formlabs Inc., Somerville, MA, USA) due to its desirable combination of toughness, heat resistance, and improved deformation behaviour compared to standard resins [29]. Notably, the Grey Pro Resin reportedly features a Heat Deflection Temperature (HDT) of 62 °C at 1.8 MPa and 78 °C at 0.45 MPa according to ASTM D 648-16, as documented by the manufacturer [29], highlighting its potential for applications demanding dimensional stability under moderate loads at elevated temperatures, aligning well with the focus of this study on room temperature performance (22 °C). This material selection facilitates the creation of intricate TPMS designs with small feature sizes while maintaining structural integrity during mechanical testing. Since the complexity of the designs is high and feature sizes are small, the samples were built directly on the build plate, as shown in Figure 1. The fabrication parameters are listed in Table 1. After fabricating, the samples were washed in isopropyl alcohol for 15 min. Then, they were post-cured for 15 min at 80 °C, as suggested by the material supplier, so that the desired mechanical properties were obtained.

### 2.2. Geometrical Characterisation

X-ray microscopy imaging, performed using ZEISS (Carl Zeiss AG, Oberkochen, Germany) Xradia 620 Versa machine sourced at the University of Maribor, Slovenia Faculty of Medicine, was employed to scan the geometry of three studied cell-size graded samples. The samples were imaged with a pixel size of 23.49 µm, image dimensions of 1004 × 1024, and a binning factor 2. A binning factor 2 was chosen to enhance the signal-to-noise ratio and optimise image clarity. X-ray microscopy generates a set of grayscale images (1018 slice images in our case) that depict the geometry of each slice. These images were processed to obtain the sample’s area, volume, and mass. Firstly, the images were subjected to intensity thresholding and contrast adjustment to clean the slices and convert them to bitwise (black and white) images. Next, the area of each slice was obtained by counting the number of white pixels, and the volume of the sample was determined by summing up the areas of all the slices and multiplying them by their half distances to neighbouring slices. The mass of the sample was then calculated by multiplying the volume with the density. The computed mass was then compared with the actual mass of the sample, and the intensity threshold and contrast adjustment were fine-tuned until the computed and actual masses matched. This approach enables the accurate determination of the slice images of the sample from the X-ray microscopy images.

### 2.3. Mechanical Characterisation

#### 2.3.1. Base Material

Experimental and computational studies were conducted to investigate the strain-rate dependency of the base material. A specially designed bulk geometry was employed to achieve this.

**Figure 2:**
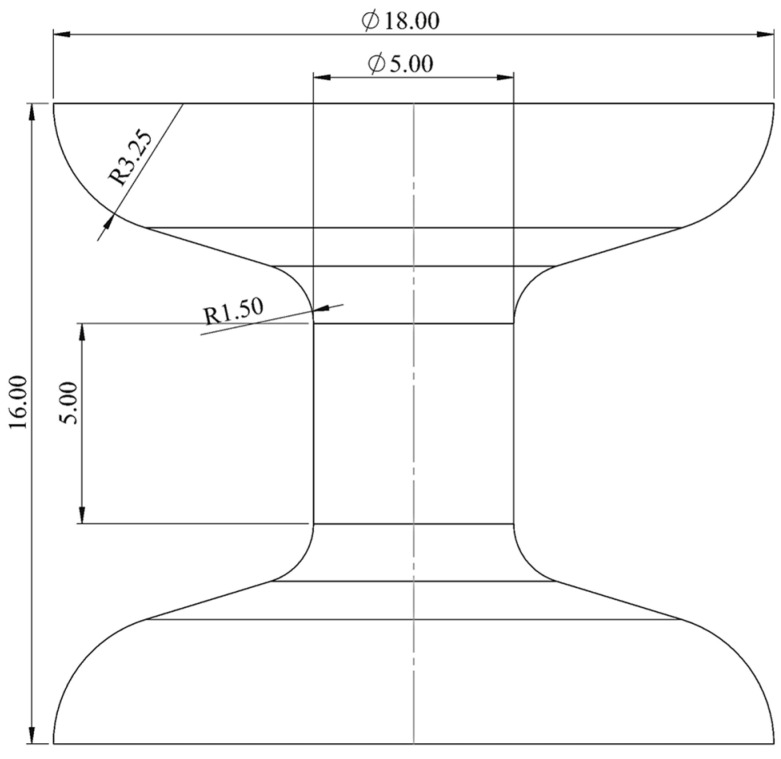
Dimensions of bulk sample.

The dimensions and geometry of the utilised bulk sample are illustrated in Figure 2. The sample exhibits large end sections to guarantee consistent contact and parallelism along the loading axis during mechanical characterisation. The sample’s central region features a uniform and smaller cross-sectional area, deliberately designed to act as the weak zone where deformation is primarily expected to occur. The transition from the sample ends to the centre is smoothly rounded to prevent stress concentration. The gauge length in the middle of the sample was 5 mm. A total of 30 bulk samples were fabricated using Stereolithography (SLA). The fabrication methodology was the same as that used to fabricate the TPMS samples, see Section 2.1.

All fabricated bulk samples were experimentally tested under compression up to 4 mm deformation (80% of the gauge length) using a Tinius Olsen H10kt testing machine (Tinius Olsen, Horsham, PA, USA) sourced from the University of Maribor, Faculty of Mechanical Engineering, Maribor, Slovenia. The tests were conducted at ten different deformation rates: 6 mm/min, 30 mm/min, 90 mm/min, 150 mm/min, 180 mm/min, 210 mm/min, and 240 mm/min, 300 mm/min, 360 mm/min, and 500 mm/min. Tests were repeated three times at each deformation rate. While deformation rates are not directly equivalent to strain rates, the given deformation rates can be directly converted to strain rates, assuming a uniform deformation and a constant gauge length for all bulk samples. The corresponding strain rates are 0.02 s^−1^, 0.1 s^−1^, 0.3 s^−1^, 0.5 s^−1^, 0.6 s^−1^, 0.7 s^−1^, 0.8 s^−1^, 1 s^−1^, 1.2 s^−1^, and 1.67 s^−1^, respectively. Conversely, for cell-graded Diamond, Gyroid, and Primitive structures, non-uniform deformation is expected, indicating that the strain rate spatially differs along the structure, and it is impossible to state the strain rate with a single value. For consistency, this article only provides the deformation rate, that is, the speed of the upper loading plate.

Although the deformation rates used in this study were relatively low, the effect of inertia was carefully quantified to verify that any observed differences in response to different deformation rates could be attributed to the strain rate sensitivity of the raw material.

#### 2.3.2. Graded TPMS Structures

To evaluate the mechanical properties of the TPMS structures at different deformation rates, three experiments were conducted for each studied graded TPMS structure at five different deformation rates: 6 mm/min, 60 mm/min, 120 mm/min, 240 mm/min, and 500 mm/min (45 experiments in total). A Tinius Olsen H10kt testing machine (Tinius Olsen, Horsham, PA, USA) sourced from the University of Maribor, Faculty of Mechanical Engineering, Maribor, Slovenia, was used. For each experiment, the stress values for each strain level were determined. Then, the average stress values were computed for each strain level from the three experiments for each topology and deformation rate. Then, the average initial peak stress, plateau stress, densification strain, and specific energy absorption were determined for each topology at each deformation speed. The initial peak stress is when the structure experiences the local maximum stress after the initial elastic response. The plateau stress is the average stress in the 20–40% compressive strain range. The densification strain is the strain at stress equal to 1.3 times the plateau stress. The specific energy absorption is calculated as follows [2,30]:

(1) $$\mathrm{SEA}=\frac{\int_{0}^{0.4}\sigma d\varepsilon }{\rho }$$

We conducted full-field deformation analyses to comprehensively understand the deformation mechanisms and the impact of cell grading on deformation fields. We used a Canon EOS R camera with an EF 100 mm f/2.8 Macro lens to capture images of the deforming TPMS structures during quasi-static compression experiments. We manually selected specific locations on the deforming TPMS structures for each experiment. Then, we tracked these locations using Digital Image Correlation (DIC). Four points were chosen from the corners of the bounding rectangle of the sample, while the other points were strategically selected from both the small and large cell sides. The main criteria for selection were to distribute the points evenly across the structure and ensure their trackability throughout the deformation process. We then calculated deformations for each tracked point and overlaid heatmaps of these deformations onto the original images to visualise and analyse the deformation patterns.

### 2.4. Computational Modelling

A computational model of the bulk material sample was developed using LS-Dyna software (The pre-processing was performed using LS-PrePost 4.8, and the post-processing utilized LS-Post 2021 R2) to capture the response of experimental testing conducted at a quasi-static loading rate (6 mm/min) and to investigate the inertial effects at higher strain rates. This allowed for a comprehensive understanding of the material’s response under various loading conditions, considering both its strain rate dependency and the impact of inertia. Importantly, no strain rate parameters were included in the simulation to isolate the effect of inertial forces. Instead, the model was run at different deformation rates to assess the influence of inertial forces directly. This approach ensured that any observed differences in response could be attributed solely to the inertial effects without the confounding influence of strain rate dependencies. An attempt was made to match the reaction forces measured with the load cell at 6 mm/min to their computational counterparts with the material constitutive parameters adjustment.

**Figure 3:**
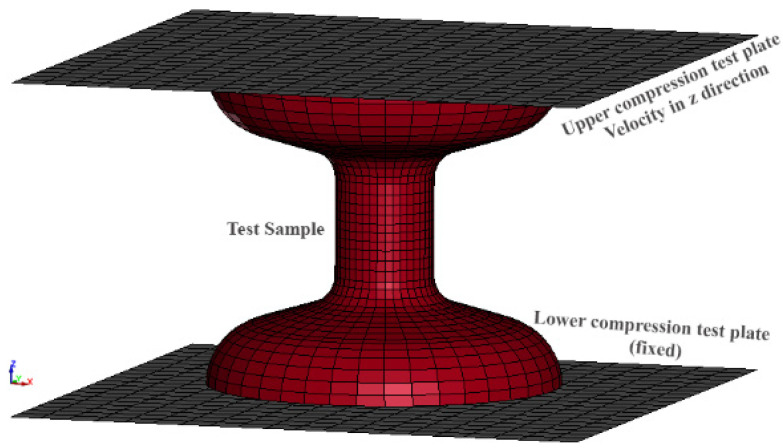
Computational model with meshed elements.

Despite the design of the bulk samples aiming to induce deformation primarily in the central, uniform zone, some deformation inevitably occurs throughout the overall geometry, even though not as pronounced. This necessitates computational modelling, as measured force/stress values cannot be directly linked to measured displacement/strain under these conditions. Computational modelling provides a detailed and accurate representation of the material’s behaviour over the entire sample. The bulk material test sample was created using 12,600 eight-point hexahedron solid elements Figure 3. The constitutive behaviour of the photopolymer base material “Grey Pro” was described using the MAT_PIECEWISE_LINEAR_PLASTICITY model (MAT_024). The material’s density was computed by dividing the known volume, as obtained from CAD, by the measured mass. Poisson’s ratio was determined experimentally by using image processing techniques. The other material parameters were obtained iteratively by minimising the discrepancy between the reaction force estimated from the computational model and experimental measurements.

The upper and lower steel plates were modelled as linear elastic materials (MAT_ELASTIC), each consisting of 800 Belytschko–Tsay shell elements with a thickness of 2 mm and a size of 30 mm × 30 mm. The material properties of the plates were set to E = 210 GPa, ν = 0.3, and ρ = 0.00785 g/mm^3^. To model the lower compression test plate, the *BOUNDARY_SPC_SET card was used to select all nodes on the plate and constrain their translational and rotational movements.

The AUTOMATIC_SURFACE_TO_SURFACE option was utilised to model the contact between the two plates and the test sample. Furthermore, static and dynamic friction coefficients of 0.2 and 0.3 were established between the plates and the test sample to account for frictional effects.

**Figure 4:**
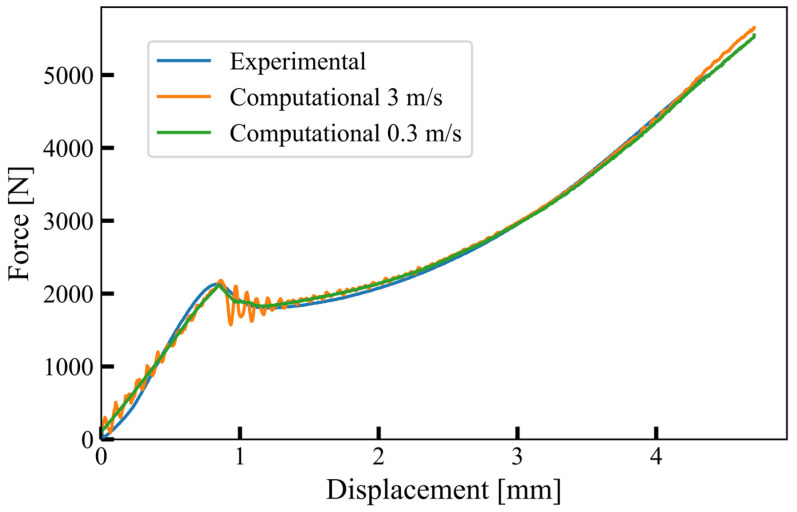
Comparison of experimental response (6 mm/min) with reaction forces obtained from top plate in computational model at 3 m/s and 0.3 m/s.

Explicit time integration schemes in computational simulations impose a constraint on the time step, determined by the minimum element size divided by the wave speed. This limitation necessitates a significant number of iterations to simulate slow quasi-static experiments, such as the one conducted in this study (approximately 30 s). To expedite the simulation, time scaling was employed to accelerate the quasi-static loading (6 mm/min) by increasing the velocity of the upper compression plate. This was achieved by applying the PRESCRIBED_MOTION_RIGID card, enabling z-translational motion for all upper plate nodes while restricting all other degrees of freedom. Careful selection of the upper plate velocity was crucial to prevent spurious effects arising from inertial forces. We initiated with a high initial velocity and iteratively decreased it until the simulated response matched the observed experimental behaviour. As the velocity decreased, oscillations in the reaction forces diminished, achieving a satisfactory response at 3 m/s. The optimal alignment with experimental results was attained at a lower velocity of 0.3 m/s, showcasing smoother reaction forces. Figure 4 illustrates the comparison of reaction force from experimental measurement and computational model.

**Figure 5:**
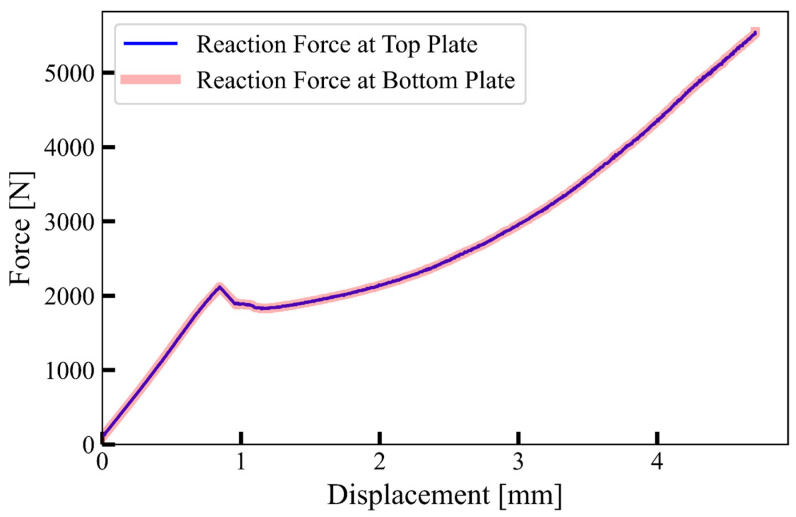
Comparison of reaction forces obtained from top plate and bottom plate in computational model at 0.3 m/s.

The deformation speed was validated by verifying the equilibrium of the reaction forces on the lower and upper plate, see Figure 5. The reaction forces exerted on the top and bottom plates were nearly identical. This indicates that using an increased upper plate velocity of 0.3 m/s, the computational model was still in quasi-static equilibrium and accurately reflected the experimental measurements at a 6 mm/min deformation rate.

## 3. Results and Discussion

### 3.1. Geometrical Characterisation

**Figure 6:**
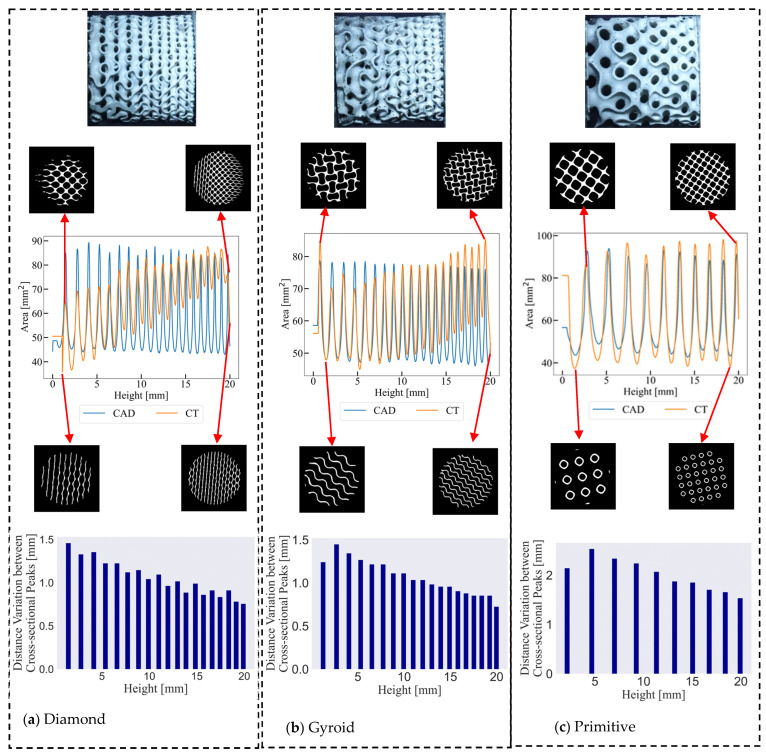
X-ray microscopy results and geometrical features of cell-size graded samples showing the surface area variation and the height variation between peaks in cross-sectional area for (**a**) Diamond, (**b**) Gyroid, and (**c**) Primitive cells.

The results of the geometrical characterisation of cell-size graded Diamond, Gyroid, and Primitive structures are shown in Figure 6. The figure clearly illustrates that the structures were designed to maintain a uniform relative density. Notably, Primitive structures exhibited thicker and simpler shells, enabling closer alignment with the intended CAD model during manufacturing. An interesting contrast emerges when evaluating the physical samples of Gyroid and Diamond structures compared to their respective CAD designs. While cell-size graded Gyroid structures align relatively closely with the CAD model, the Diamond structures exhibit notable deviations. It is worth noting that Diamond and Gyroid structures unexpectedly resulted in thicker shells within the small cell region than were intended by the CAD model. This discrepancy highlights a clear limitation within the manufacturing process, particularly in fabricating Diamond structures. Moreover, it is important to emphasise that cell-size graded Gyroid and Diamond structures share the characteristic of thinner shells adorned with sophisticated details. Of particular interest is the observation that the distance between cross-sectional peaks diminishes as one transitions toward regions characterised by smaller cellular configurations. This looming trend corresponds with the anticipated proximity of small unit cells and connected regions within these areas. Importantly, this pattern suggests an influence on the deformation behaviour of the structures.

### 3.2. Mechanical Characterisation

#### 3.2.1. Base Material

**Figure 7:**
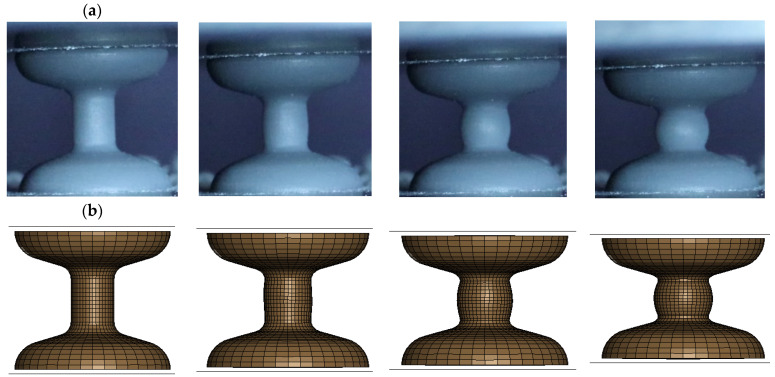
Deformation behaviour of bulk samples: (**a**) real experiment at 6 mm/min; (**b**) computational model (0.3 m/s).

Figure 7 indicates the deformation behaviour of bulk samples in the experiment and computational model.

Table 2 lists computationally iteratively determined material model parameters for the photopolymer base “Grey Pro”.

**Figure 8:**
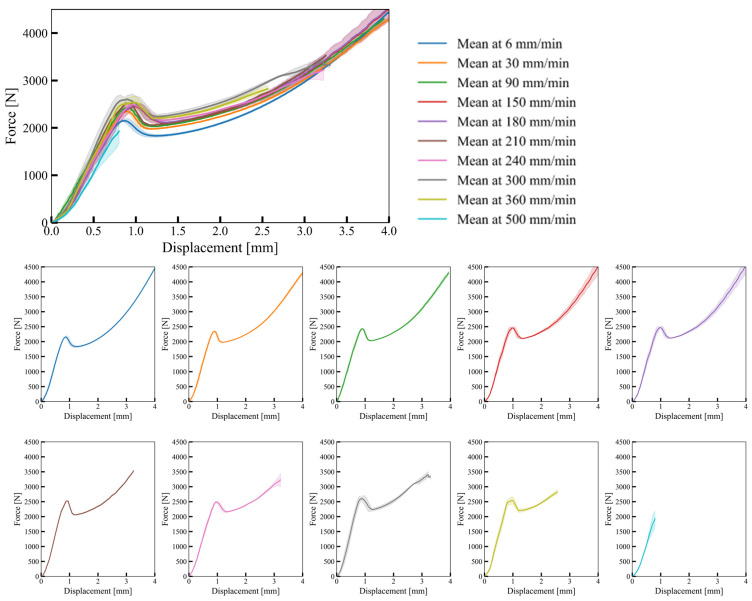
Force–displacement relationships of bulk material samples at different deformation rates (combined upper, separate experimental data lower). The shaded area around the mean force-displacement curve indicates the standard deviation of the response for a given deformation rate.

Before addressing the experimental response, it is important to note that the deformation rates employed in the computational simulations were significantly greater than those of the actual experiment (6 mm/min). Despite this tenfold increase in velocity (3 m/s and 0.3 m/s), the reaction force curve’s overall trend remained unchanged, with only minor fluctuations observed (refer to Figure 4). This suggests that inertial forces had minimal impact on the overall reaction force response trend when the deformation rate is 0.3 m/s, despite this loading rate being 3000 times faster than the experiment’s. Notably, Figure 8 depicts a substantial change in the bulk material’s response at varying deformation rates, indicating that the observed increase is primarily attributed to the strain rate dependency of the base material with minimal inertial effects.

**Figure 9:**
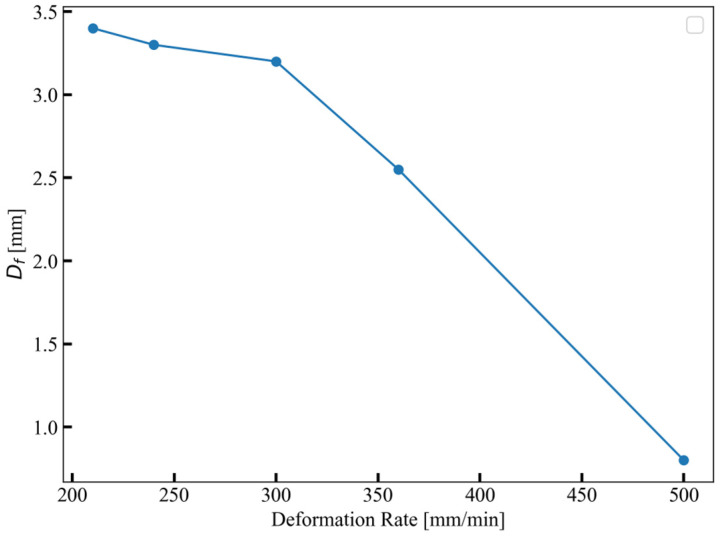
Initiation of failure deformation (D_f_) at varying strain rates.

As mentioned earlier, three separate experiments were conducted at each deformation rate. For slow deformation rates up to 180 mm/min, no failure was observed until a deformation of 4 mm was reached. However, in one out of three experiments conducted at faster deformation rates of 210 mm/min and 240 mm/min, failure occurred before reaching 4 mm. Further increasing the deformation rate to 300 mm/min, 360 mm/min, and 500 mm/min resulted in even earlier failure initiation, with all samples failing before 4 mm. The experimental compression tests identified a critical deformation rate of 210 mm/min corresponding to a strain rate of 0.7 s^−1^, signifying a shift in the material’s susceptibility to failure. The average failure deformation for each deformation rate after the critical deformation rate is provided in Figure 9.

#### 3.2.2. Graded TPMS Structures

**Figure 10:**
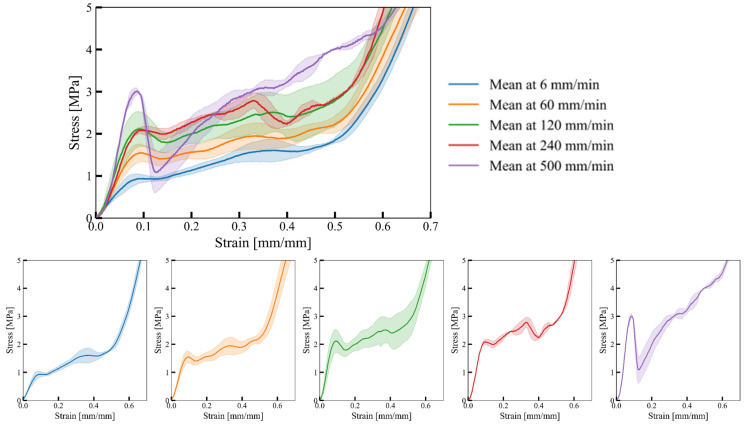
Mechanical response of cell-size graded Diamond structure at different deformation rates. The shaded area around the mean stress–strain curve indicates the standard deviation of the response for a given deformation rate.

The experimentally determined mechanical response of cell-size graded Diamond structures in terms of the engineering stress–strain relationship is shown in Figure 10.

**Figure 11:**
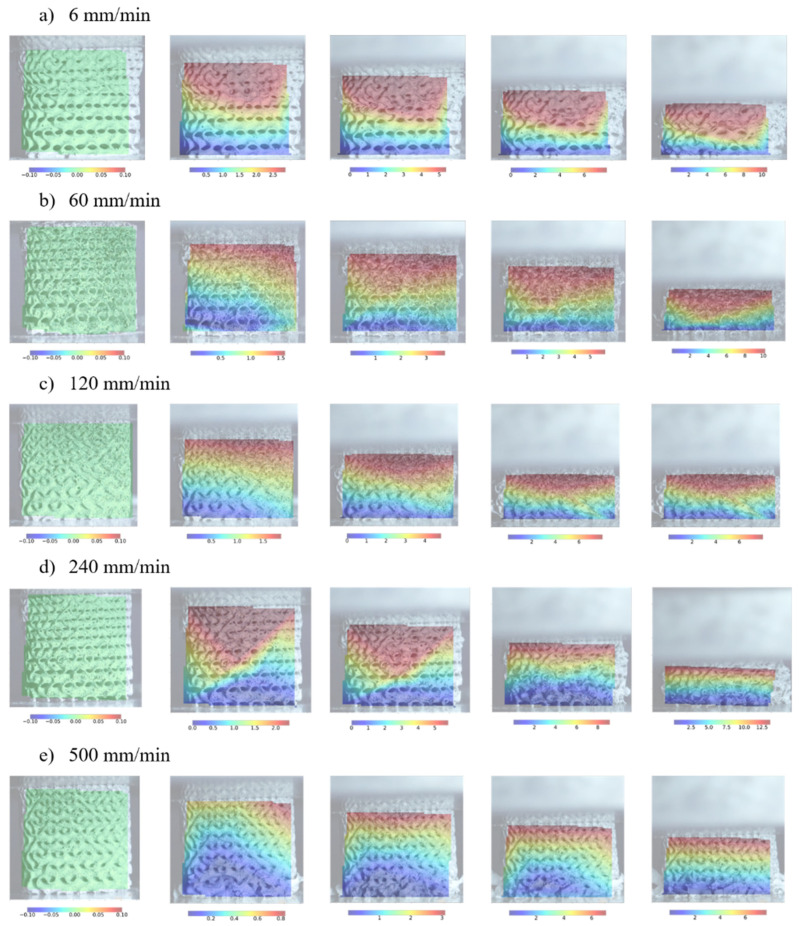
Deformation process of cell-size graded Diamond structures at different deformation rates.

The results demonstrate that cell-size graded Diamond structures exhibit typical cellular material behaviour. Following the initial peak stress, a noticeable drop in strength is attributed to the collapse of cell walls, which becomes more pronounced with increasing deformation rates. As deformation progresses, further collapse of cell walls occurs, leading to contact between adjacent cell walls and eventual densification of the structure. Notably, deformation rates of 500 mm/min and 240 mm/min exhibit significant differences compared to lower strain rates, primarily due to early failure of cell walls rather than deformation. Furthermore, across all structures, most deformation initiates in regions of smaller cells with lower stiffness (Figure 11), triggering the initial collapse. Subsequently, deformation propagates to the larger cell region. At a deformation rate of 500 mm/s, we observed that small cells undergo ductile deformation, while large cells are susceptible to failure characterised by sudden cracks. This indicates a non-homogeneous strain rate due to the non-homogeneous deformation regime, where the strain rate in the failure region exceeds the critical strain rate.

**Figure 12:**
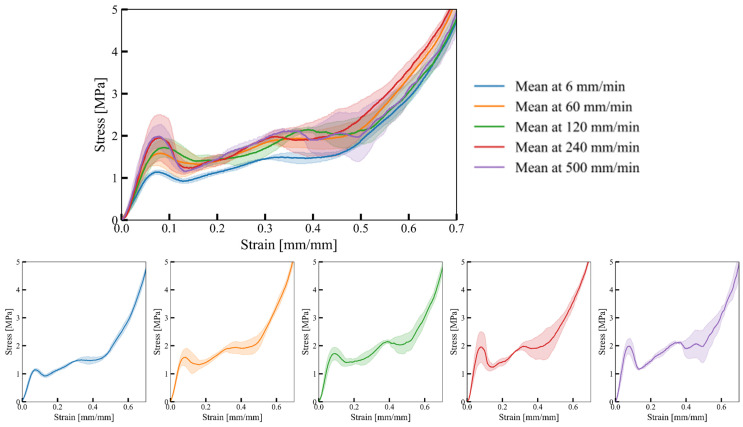
Mechanical response of cell-size graded Gyroid structure at different deformation rates. The shaded area around the mean stress–strain curve indicates the standard deviation of the response for a given deformation rate.

The experimentally determined mechanical response of cell-size graded Gyroid structures in terms of engineering stress–strain relationship is shown in Figure 12.

**Figure 13:**
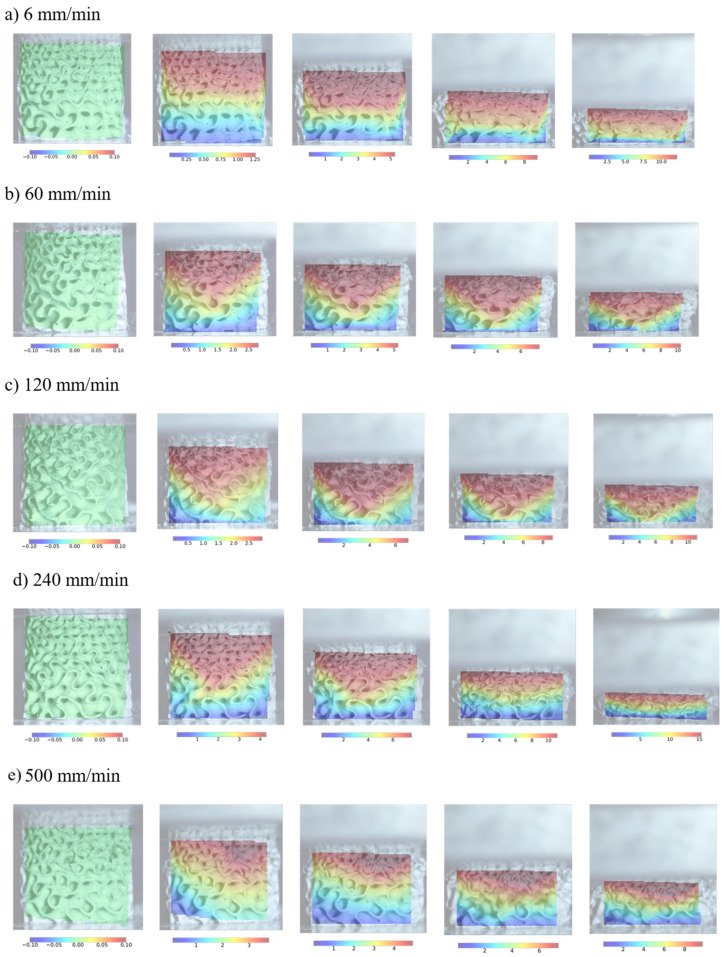
Deformation process of cell-size graded Gyroid structures at different deformation rates.

The results demonstrate that cell-size graded Gyroid structures exhibit similar cellular material mechanical properties to cell-size graded Diamonds, including strain rate sensitivity. An increasing deformation rate causes an increase in the initial peak stress. After reaching the initial peak stress, a decrease in stress is observed due to the subsequent collapse of cell walls. The drop is more prominent at higher strain rates. As deformation progresses, cell walls continue to collapse, leading to contact between adjacent cell walls and, ultimately, densification of the structure. Significant deviations in the response are observed at high deformation rates of 240 mm/min and 500 mm/min due to the early failure of cell walls. Figure 13 shows the structure deformation process overlaid with the full-field strain field.

The results show that most deformation occurs in regions of smaller cells with lower stiffness, where the initial collapse is initiated. Once the smaller cells fully collapse, the deformation propagates to the larger cell region. At 240 mm/s and 500 mm/s deformation rates, we observed that small cells experience ductile deformation, while large cells are susceptible to brittle failure, which is characterised by sudden cracks.

**Figure 14:**
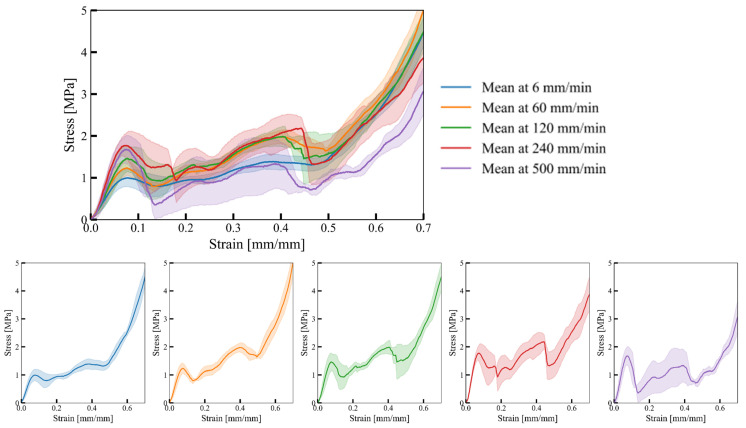
Mechanical response of cell-size graded Primitive structure at different deformation rates. The shaded area around the mean stress–strain curve indicates the standard deviation of the response for a given deformation rate.

The experimentally determined mechanical response of cell-size graded Primitive structures in terms of the engineering stress–strain relationship is shown in Figure 14.

The initial response of cell-graded Primitives is similar to that of cell-size graded Diamonds and Gyroids. As the deformation rate increases, the collapse initiation stress also increases. After reaching the initial peak stress, a noticeable decrease in stress is observed due to the collapse of cell walls. This drop is again more prominent at higher strain rates. As deformation progresses, cell walls collapse, leading to contact between adjacent cell walls and increased mechanical strength. A drop in stress is observed at around 0.4 strain due to the gradual collapse initiation of large cells (Figure 14).

**Figure 15:**
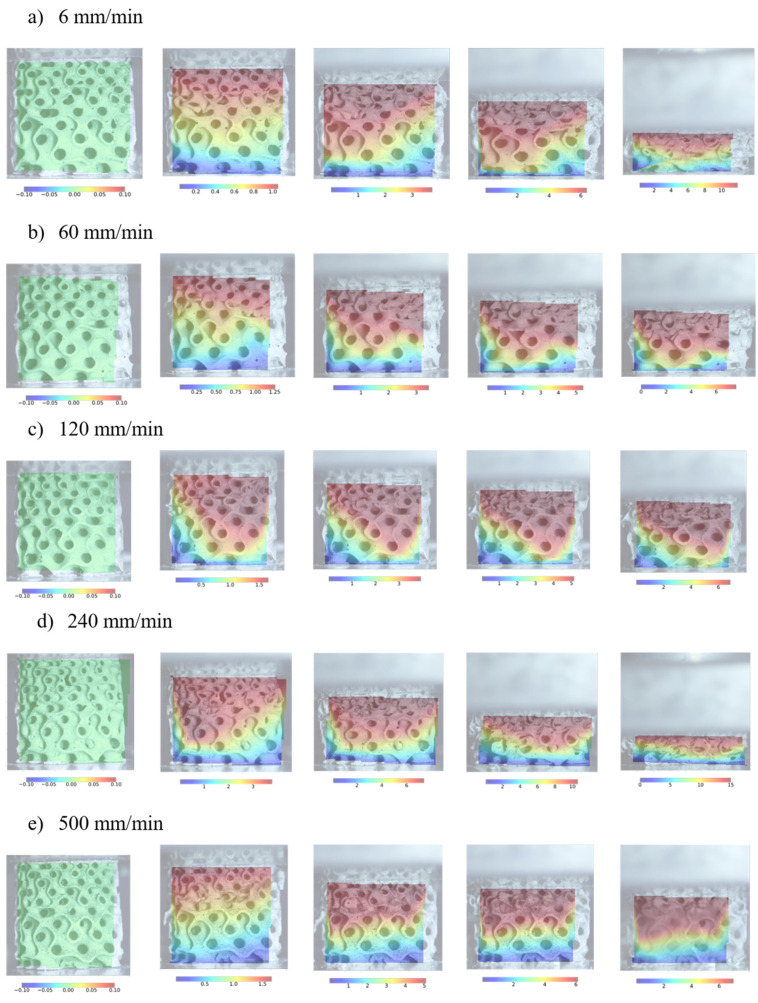
Deformation process of cell-size graded Primitive structures at different deformation rates.

Figure 15 shows the structure deformation process overlaid with the full-field strain field.

The compliance of the small cell sides suggests they undergo more deformation than their larger counterparts. Furthermore, large deformation causes the formation of shear zones at the small cell sides. The observations reveal that failure initiates and propagates at the shells from the outer region due to stress concentrations at the edges of the structure. The large difference in stiffness results in the large cell sides deforming only after almost complete deformation of small cells. Again, an early failure of large cells was observed at 500 mm/s deformation rate.

The investigation showed that small cell sides exhibit higher compliance than large cell sides. Additionally, initial elastic deformation at the small side samples continues with the shear zone until collapse. The collapse begins with small cells and progresses to larger ones, which collapse fully before complete densification. Notably, at a deformation rate of 500 mm/min, all samples failed in a brittle manner from the bottom (large cell direction).

**Figure 16:**
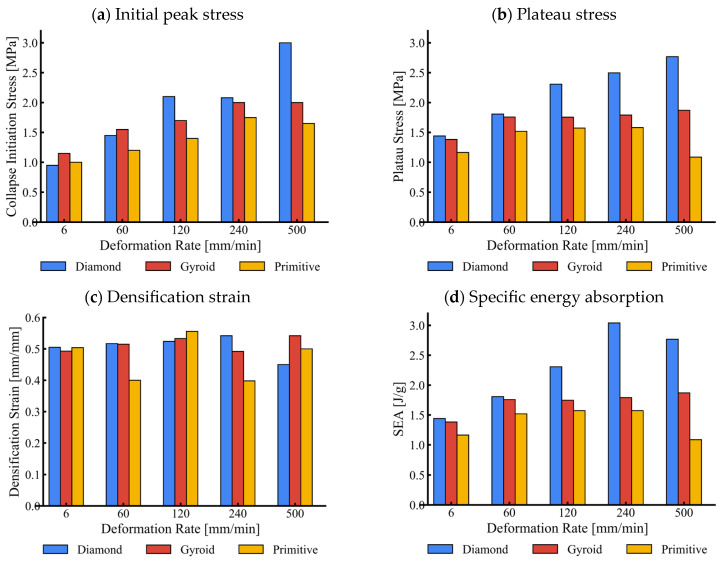
Comparison of cell-graded Diamond, Gyroid, and Primitive structures’ mechanical parameters at various deformation rates.

Figure 16 compares the mechanical parameters of analysed TPMS structures at various deformation rates. It can be observed that mechanical parameters generally increase with the increase in the deformation rate for all analysed TPMS structures due to the strain rate dependency of the base material. The inertial effects are insignificant at the deformation rates considered in this study, as discussed in Section 3.2.1.

However, the plateau stress and, consequently, the SEA reduce at the highest deformation speed of 500 mm/min in cell-size graded Primitive structures, which is caused by earlier failure of the base material at this deformation rate. The cell-size graded Diamond structure has the highest energy absorption capability due to its increased stiffness resulting from base cell geometry.

## 4. Conclusions

The study presents investigation results of the mechanical behaviour of three different TPMS structures (Diamond, Gyroid, and Primitive) made of thermoset plastics (photopolymer base “Grey Pro”). The study aimed to isolate the individual effects of base material, inertia, topology, and cell grading on the mechanical properties of TPMS structures by conducting both experimental and computational investigations at different strain rates. Actual connectivity of the walls was observed using an X-ray microscopy machine to understand the topology properties better. A full-field deformation analysis based on digital image correlation was used to examine non-homogeneous deformation.

Analysing the mechanical response of cell-size graded TPMS structures with their complex geometries is a daunting challenge. The deformation mechanism changes dynamically during densification, and the thinner and longer cell walls are more flexible and prone to buckling and bending. However, these structures exhibit two distinct regions, one with thinner and shorter walls and the other with thicker and longer shells, making predicting their mechanical performance in advance difficult.

To understand the strain rate dependency of the base material and isolate it from inertial effects, we conducted both experimental and computational studies. Our findings reveal that the base material strengthens as the strain rate increases and the inertial effects are minimal at studied deformation rates.

Our study identified a critical strain rate 0.7 s^−1^ for the photopolymer “Grey Pro” base material, where the base material became more susceptible to failure as the deformation and strain rate increased.

In our detailed image processing-based deformation analysis of all studied TPMS structures, we observed highly non-homogeneous deformation behaviour, resulting in substantial spatial variations. While we conducted a comprehensive deformation analysis, including detailed investigations of displacement and shape changes, we did not perform specific strain and strain rate analyses. Such analyses would require a detailed assessment of the connectivity of tracked points and the application of formulations derived from finite strain theory. However, this level of analysis was not conducted within the scope of this study. At lower deformation rates, the smaller cell sides exhibited higher compliance and initiated deformation. As deformation progressed, the cell walls contacted each other, enhancing the stiffness of the smaller cell region. In Primitive cells, the stiffness difference between the smaller and larger cell regions was so significant that the larger cell regions deformed and collapsed after full densification of the smaller cell region.

At a deformation rate of 240 mm/min and 500 mm/min, some regions of the TPMS structures experienced a higher strain rate than the critical strain rate range, resulting in a brittle failure.

We observed that the collapse initiation stress, plateau stress, densification strain, and specific energy absorption increase with strain rates for all tested TPMS structures below the critical strain rate range. However, a substantial reduction in plateau and specific energy absorption was observed for cell-graded Primitive structures above the critical strain rate range.

This study offers valuable insights into the influence of cell size and deformation rate on the mechanical behaviour of photopolymer Triply Periodic Minimal Surface (TPMS) structures. However, there are limitations to consider when interpreting the results:

Long-term behaviour: The current study investigates the mechanical response under short-term deformation conditions. The long-term behaviour of the TPMS structures under sustained loads or environmental factors is not explored and requires further investigation.

Viscoelastic Properties: While this study focuses on the mechanical behaviour at room temperature (22 °C), the material’s viscoelastic properties and behaviour across a broader temperature range are important considerations. Techniques like Dynamic Mechanical Analysis (DMA) could provide valuable information on these aspects. However, due to limitations in available instrumentation, such analyses were not performed in this study.

The manufacturer-provided HDT data [29] (62 °C at 1.8 MPa and 78 °C at 0.45 MPa according to ASTM D 648-16) offers a preliminary reference for understanding how temperature influences the stress required to deform the material by a specific amount.

## Figures and Tables

**Table 1:** Stereolithography fabrication parameters.

Machine	Form 2 from Formlabs
Material	Grey Pro from Formlabs [29]
Layer Thickness [μm]	50
Spot Size [μm]	140
Power [mW]	96
Material Volume [mL]	970 (Diamond)—1000 (Gyroid)—1030 (Primitive)
Washing Time with IPA [min.]	15
Curing Time [min.]	15
Curing Temperature [°C]	80

**Table 2:** The MAT_024 material model parameters determined for photopolymer base material “Grey Pro”.

*ρ* [kg/m^3^]	*E* [MPa]	*ν* [-]	*C* [MPa]	*P* [MPa]
1080	1240	0.4	0	0
*σ*_1_ [MPa]	*σ*_2_ [MPa]	*σ*_3_ [MPa]	*σ*_4_ [MPa]	*σ*_5_ [MPa]
104	77	78	82	122
*ε*_pl,1_ [-]	*ε*_pl,2_ [-]	*ε*_pl,3_ [-]	*ε*_pl,4_ [-]	*ε*_pl,5_ [-]
0	0.07	0.26	0.45	0.68

where *ρ*: mass density; *E*: elastic modulus; *ν*: Poisson’s ratio; *C* and *P*: strain rate parameters; *σ*, *ε*_pl_: corresponding plastic stress and strain parameters.

## Data Availability

Data are contained within the article.

## References

[1] Novak N., Vesenjak M., Duarte I., Tanaka S., Hokamoto K., Krstulović-Opara L., Guo B., Chen P., Ren Z. (2019). Compressive behaviour of closed-cell aluminium foam at different strain rates. Materials.

[2] Novak N., Al-Ketan O., Mauko A., Yilmaz Y.E., Krstulović-Opara L., Tanaka S., Hokamoto K., Rowshan R., Abu Al-Rub R.K., Vesenjak M. (2023). Impact loading of additively manufactured metallic stochastic sheet-based cellular material. Int. J. Impact Eng..

[3] Thomas T., Tiwari G. (2019). Crushing behavior of honeycomb structure: A review. Int. J. Crashworthiness.

[4] Al-Ketan O., Lee D.W., Rowshan R., Abu Al-Rub R.K. (2020). Functionally graded and multi-morphology sheet TPMS lattices: Design, manufacturing, and mechanical properties. J. Mech. Behav. Biomed. Mater..

[5] Zhang L., Feih S., Daynes S., Chang S., Wang M.Y., Wei J., Lu W.F. (2018). Energy absorption characteristics of metallic triply periodic minimal surface sheet structures under compressive loading. Addit. Manuf..

[6] Sengsri P., Fu H., Kaewunruen S. (2022). Mechanical Properties and Energy-Absorption Capability of a 3D-Printed TPMS Sandwich Lattice Model for Meta-Functional Composite Bridge Bearing Applications. J. Compos. Sci..

[7] Shi X., Liao W., Li P., Zhang C., Liu T., Wang C., Wu J. (2020). Comparison of Compression Performance and Energy Absorption of Lattice Structures Fabricated by Selective Laser Melting. Adv. Eng. Mater..

[8] Novak N., Vesenjak M., Ren Z. (2017). Computational Simulation and Optimization of Functionally Graded Auxetic Structures Made From Inverted Tetrapods. Phys. Status Solidi B Basic Res..

[9] Xu F., Zhang X., Zhang H. (2018). A review on functionally graded structures and materials for energy absorption. Eng. Struct..

[10] Zhou J., Guan Z.W., Cantwell W.J. (2013). The impact response of graded foam sandwich structures. Compos. Struct..

[11] Cui L., Kiernan S., Gilchrist M.D. (2009). Designing the energy absorption capacity of functionally graded foam materials. Mater. Sci. Eng. A.

[12] Zhang X., Zhang H. (2013). Optimal design of functionally graded foam material under impact loading. Int. J. Mech. Sci..

[13] Yu S., Sun J., Bai J. (2019). Investigation of functionally graded TPMS structures fabricated by additive manufacturing. Mater. Des..

[14] Zhang C., Jiang Z., Zhao L., Guo W., Jiang Z., Li X., Chen G. (2021). Mechanical characteristics and deformation mechanism of functionally graded triply periodic minimal surface structures fabricated using stereolithography. Int. J. Mech. Sci..

[15] Sun Y., Li Q.M. (2018). Dynamic compressive behaviour of cellular materials: A review of phenomenon, mechanism and modelling. Int. J. Impact Eng..

[16] Karimipour-Fard P., Behravesh A.H., Jones-Taggart H., Pop-Iliev R., Rizvi G. (2020). Effects of design, porosity and biodegradation on mechanical and morphological properties of additive-manufactured triply periodic minimal surface scaffolds. J. Mech. Behav. Biomed. Mater..

[17] Ruan D., Lu G., Ong L.S., Wang B. (2007). Triaxial compression of aluminium foams. Compos. Sci. Technol..

[18] Galehdari S.A., Kadkhodayan M., Hadidi-Moud S. (2015). Low velocity impact and quasi-static in-plane loading on a graded honeycomb structure; experimental, analytical and numerical study. Aerosp. Sci. Technol..

[19] Song B., Chen W., Lu W.Y. (2007). Compressive mechanical response of a low-density epoxy foam at various strain rates. J. Mater. Sci..

[20] Idris M.I., Vodenitcharova T., Hoffman M. (2009). Mechanical behaviour and energy absorption of closed-cell aluminium foam panels in uniaxial compression. Mater. Sci. Eng. A.

[21] Abate K.M., Nazir A., Yeh Y.P., Chen J.E., Jeng J.Y. (2020). Design, optimization, and validation of mechanical properties of different cellular structures for biomedical application. Int. J. Adv. Manuf. Technol..

[22] Szatkiewicz T., Laskowska D., Bałasz B., Mitura K. (2022). The Influence of the Structure Parameters on the Mechanical Properties of Cylindrically Mapped Gyroid TPMS Fabricated by Selective Laser Melting with 316L Stainless Steel Powder. Materials.

[23] Wu Y., Qiao D., Tang L., Xi H., Liu Y., Jiang Z., Liu Z., Zhou L. (2019). Global topology of failure surfaces of metallic foams in principal-stress space and principal-strain space studied by numerical simulations. Int. J. Mech. Sci..

[24] Miralbes R., Ranz D., Pascual F.J., Zouzias D., Maza M. (2022). Characterization of additively manufactured triply periodic minimal surface structures under compressive loading. Mech. Adv. Mater. Struct..

[25] Kucewicz M., Baranowski P., Stankiewicz M., Konarzewski M., Płatek P., Małachowski J. (2019). Modelling and testing of 3D printed cellular structures under quasi-static and dynamic conditions. Thin-Walled Struct..

[26] Wang E., Chen C., Zhang G., Luo Q., Li Q., Sun G. (2023). Multiaxial mechanical characterization of additively manufactured open-cell Kelvin foams. Compos. Struct..

[27] Al-Ketan O., Abu Al-Rub R.K. (2021). MSLattice: A free software for generating uniform and graded lattices based on triply periodic minimal surfaces. Mater. Des. Process. Commun..

[28] Novak N., Al-Ketan O., Krstulović-Opara L., Rowshan R., Abu Al-Rub R.K., Vesenjak M., Ren Z. (2021). Quasi-static and dynamic compressive behaviour of sheet TPMS cellular structures. Compos. Struct..

[29] “Grey-Pro”. https://formlabs.com/eu/store/materials/grey-pro-resin/.

[30] (2011). Mechanical Testing of Metals—Ductility Testing—Compression Test for Porous and Cellular Metals.

